# A data fusion approach for nondestructive tracking of the ripening process and quality attributes of green Hayward kiwifruit using artificial olfaction and proximal hyperspectral imaging techniques

**DOI:** 10.1002/fsn3.3548

**Published:** 2023-07-06

**Authors:** Adel Bakhshipour

**Affiliations:** ^1^ Department of Biosystems Engineering, Faculty of Agricultural Sciences University of Guilan Rasht Iran

**Keywords:** data fusion, electronic nose, machine learning, nondestructive methods, quality attributes, spectral reflectance

## Abstract

A data fusion strategy based on hyperspectral imaging (HSI) and electronic nose (e‐nose) systems was developed in this study to inspect the postharvest ripening process of Hayward kiwifruit. The extracted features from the e‐nose and HSI techniques, in single or combined mode, were used to develop machine learning algorithms. Performance evaluations proved that the fusion of olfactory and reflectance data improves the performance of discriminative and predictive algorithms. Accordingly, with high classification accuracies of 100% and 94.44% in the calibration and test stages, the data fusion‐based support vector machine (SVM) outperformed the partial least square discriminant analysis (PLSDA) for discriminating the kiwifruit samples into eight classes based on storage time. Moreover, the data fusion‐based support vector regression (SVR) was a better predictor than partial least squares regression (PLSR) for kiwifruit firmness, soluble solids content (SSC), and titratable acidity (TA) measures. The prediction *R* ^2^ and RMSE criteria of the SVR algorithm on the test data were 0.962 and 0.408 for firmness, 0.964 and 0.337 for SSC, and 0.955 and 0.039 for TA, respectively. It was concluded that the hybrid of e‐nose and HSI systems coupled with the SVM algorithm delivers an effective tool for accurate and nondestructive monitoring of kiwifruit quality during storage.

## Graphical abstract

A data fusion, based on hyperspectral imaging (HSI) and electronic nose (e‐nose) systems, was performed in this study to inspect the postharvest ripening process of Hayward kiwifruit. Performance evaluations proved that the fusion of olfactory and reflectance data improves the performance of machine learning algorithms. The data fusion‐based SVM discriminated the kiwifruit samples based on storage time with a classification accuracy 94.44% in the test stage. The SVR model predicted the kiwifruit firmness, SSC, and TA with the *R* ^2^ values of 0.962, 0.964, and 0.955, respectively. It was concluded that the hybrid of e‐nose and HSI systems coupled with the SVM algorithm delivers an effective tool for accurate and nondestructive monitoring of kiwifruit quality during storage.

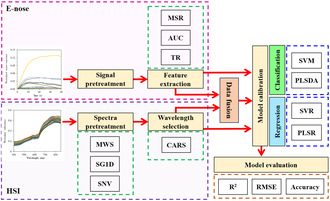

## 1 INTRODUCTION

Kiwifruit (*Actinidia deliciosa*) is becoming an economically strategic fruit worldwide. The pleasant taste, nutritional characteristics, and medicinal benefits of this subtropical product have caused more and more desire for this fruit in recent years. The area of kiwifruit orchards increased from 175,800 ha in 2011 to more than 286,900 ha in 2021. Kiwifruit production has increased from about 2.91 megatons to more than 4.47 megatons in this period (FAOSTAT, 2023). Iran is the fifth producer of kiwifruit in the world, with approximately 294,000 tons of total kiwifruit production in 2021 (FAOSTAT, 2023). The Hayward cultivar (green kiwifruit) is the most commercially common variety in Iran (Maghdouri et al., 2021), and in the world (Hussain et al., 2021).

One of the essential issues related to kiwifruit production is fruit softening and possible rotting in the postharvest process. During the transition from maturity to edibility in the ripening process, physiochemical reactions increase the amount of simple sugars in the fruit. In addition, change in the fruit cell walls structure during ripening causes the flesh softening of the fruit (Tangpao et al., 2022; Wang et al., 2021).

Proper monitoring of the fruit ripening process can help better manage the fruit storage process and prevent economic losses. Furthermore, determining the kiwifruit ripeness level and applying it as a grading parameter has a positive impact on its market value. The three essential postharvest attributes of Hayward kiwifruits that are effective on consumer acceptance are firmness, sweetness, and acidity (Burdon et al., 2004; Marsh et al., 2004). Sweetness of kiwifruit is mainly described by soluble solids content (SSC), and the fruit acidity is indicated by titratable acidity (TA) (Du, Xu, et al., 2019). The SSC and TA measures are highly associated with consumer preference (Han et al., 2019; Liao et al., 2022), and used in describing the taste and flavor of kiwifruits (Zhang et al., 2021). Measurement of SSC and TA is currently done by laboratory and instrumental methods. These methods provide accurate data but are almost expensive, require trained personnel, and are destructive. Hand pressure testing method is commonly used to separate the soft fruits, which causes bruising damage to the fruits. Therefore, there is a need for accurate, reliable, and nondestructive ways to monitor the kiwifruit ripening stage and its quality characteristics.

Determining the ripeness of kiwifruit by trusting only in appearance characteristics such as size, shape, and color of the fruit is challenging (Du, Wang, et al., 2019). Fortunately, there are other potential indicators instead of visible features. A critical parameter highly influenced by the fruit ripening process is the aroma emitted from the fruit (Tiwari et al., 2020). The fruit aroma depends on its volatile organic compounds (VOCs) composition, which continuously changes during the ripening process (Taiti et al., 2015).

An electronic nose (e‐nose) or artificial olfactory system is an aroma recognition method using a semiselective gas‐sensitive sensor array and signal manipulation algorithms. Successful applications of e‐noses have been reported in agricultural and food products (Ali & Hashim, 2023; Ali et al., 2020; Jia et al., 2019; Seesaard et al., 2022). An e‐nose system with five metal oxide semiconductor (MOS) sensors was developed by Tyagi et al. (2023) to classify the apple, banana, orange, grape, and pomegranate fruits into three different degrees of ripeness, namely unripe, ripe, and overripe. The classification accuracy of artificial neural networks (ANN) algorithm was more than 95%.

Hyperspectral imaging (HSI) is another promising technology in agrofood‐related quality evaluation studies. Spectral reflectances recorded from the fruit skin are highly dependent on the physical and biochemical properties and quality of fruit (Matteoli et al., 2016). The spectral reflectance of Bananito fruit peel in the range of 400–740 nm was used for nondestructive fruit classification into three ripening levels using a partial least square discrimination analysis (PLSDA) model with a classification accuracy of 93.3% (Pu et al., 2019). In a recent study, Benelli et al. (2022) investigated the capability of 400–1000 nm hyperspectral data for predicting the variations of SSC and flesh firmness values of Hayward kiwifruit samples during the ripening process using the PLS model. The best obtained prediction *R* ^2^ and RMSE values were 0.94 and 0.73 for SSC and 0.92 and 9.87 for firmness, respectively. The highest PLSDA accuracy of 97% was reported for classifying kiwifruit samples into three ripeness stages.

Data fusion is a machine learning strategy of mixing data from different analytical sources that can complete the information. Therefore, a higher modeling performance can be obtained rather than the data from single sources (Alamar et al., 2020). The concept and different applications of the data fusion approach in foods are well described in a book chapter provided by Biancolillo et al. (2019). An integration of hyperspectral and olfactory sensors coupled with multivariate data analysis algorithms was used for the quality classification of green tea according to the human panel test. The support vector machine (SVM) classifier with the fusion of spectral and olfactory data obtained a classification accuracy of 92%, which outperformed the individual sensor data with the highest accuracy of 78% (Li, Xie, et al., 2019). A fusion of hyperspectral and e‐nose data was used for nondestructive identification of microbial content and quality characteristics of strawberries during the gray mold contamination. Higher capability and superiority of the data fusion‐based model over the single dataset‐based models were also reported in this study (Liu et al., 2019).

As far as we have reviewed in previous literature, little research has been done on the use of the combination of reflectance and odor‐extracted data to evaluate the quality of fruits. Moreover, there is no research on the fusion of e‐nose and HSI systems to track the kiwifruit postharvest ripening process. Therefore, this study aimed to perform a comparative investigation on the capability of spectral reflectance and olfactory data in individual and fused modes to determine the ripening stage and predict the quality attributes of kiwifruit.

## 2 MATERIALS AND METHODS

### 2.1 Sample preparation

The experiments were conducted in a completely randomized design. In September 2021, the required fresh kiwifruit samples for this research were harvested from one tree in a commercial Hayward kiwifruit orchard around Rasht city (37.195375, 49.643989) of Guilan Province, northern Iran. The samples were carefully picked up from the tree without any damage or defect. They were carefully transferred to the postharvest technology laboratory of the Department of Biosystems Engineering, Faculty of Agricultural Sciences, University of Guilan. A total number of 120 kiwifruit samples with almost the same size and firmness were carefully selected. The surfaces of the fruits were rinsed with tap water and dried at room temperature to avoid contamination. The samples were then completely randomly divided into eight groups of 15 fruits. Therefore, there was uniformity between and within groups. The kiwifruits were kept in an incubator (WTW, TS608/G/21, Germany) at a temperature of 20°C and relative humidity of 70% to allow the natural ripening process.

The experiments, including the measurement of physiochemical attributes and the extraction of hyperspectral and e‐nose data, were carried out on eight different days (0, 2, 5, 8, 11, 14, 17, and 20 days). On each experiment day, the data were acquired from 15 kiwifruit samples.

### 2.2 Measurement of quality attributes

In this research, the variations of three essential quality parameters in kiwifruit, including flesh firmness, SSC, and TA, were monitored during the ripening process. To determine the firmness of the fruits, a penetrometer (Effegi, Model FT 011, USA) having a tip of 8‐mm diameter was used. The readings at two opposed sides along the fruit's equator after 2‐mm skin removal were averaged and expressed as Newton (N) (Feng et al., 2002; Wu et al., 2013). The fruit extract was obtained by cutting the fruit in half and then squeezing the halves of the fruit. The juices extracted from two halves of the fruit were mixed. The juice was poured on a digital refractometer (Euromex RD 635, the Netherlands) to determine the fruit SSC value in °Brix (%) (Asiche et al., 2018). The fruit juice was also titrated against 0.1 M NaOH, where phenolphthalein was employed as the pH marker (Grasso et al., 2022), to calculate the TA of kiwifruit samples as the percentage of citric acid.

### 2.3 Nondestructive tests

**FIGURE 1:**
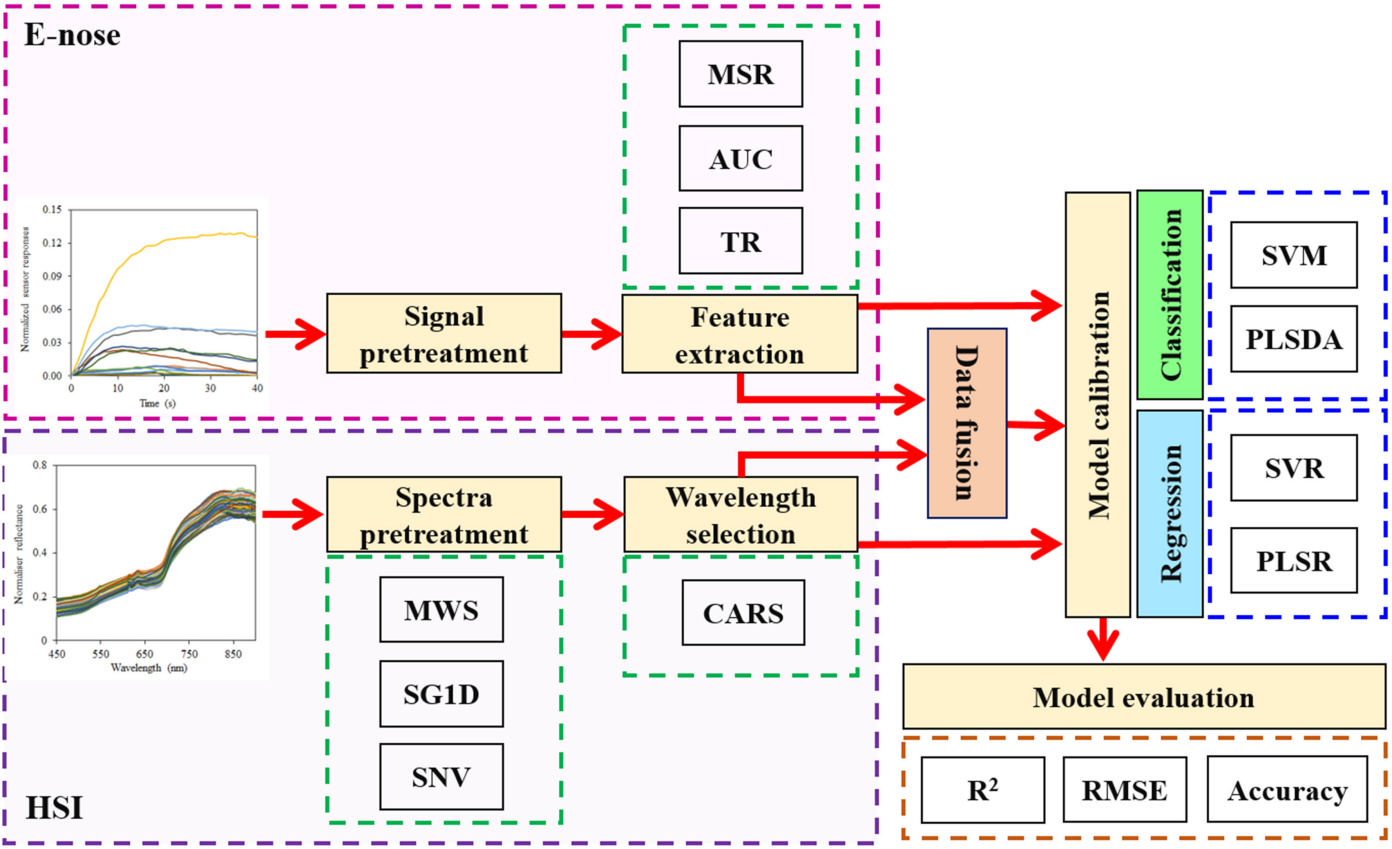
Overview diagram of the combined e‐nose and hyperspectral systems.

In this study, two nondestructive methods of e‐nose and HSI systems were used to collect data from kiwifruit samples during storage. The flow diagram of the operations performed in this research is presented in Figure 1, and the corresponding explanations are available in the following subsections. First, the e‐nose data were extracted from the single kiwifruit samples. The recorded signals were preprocessed, and the desired features were calculated from the normalized signals.

In addition to that, the surface reflectances of the kiwifruit samples were also captured. The recorded spectra were preprocessed and the effective wavelengths were selected by applying the CARS wavelength selection algorithm.

Subsequently, the classification and prediction algorithms were developed using the individual and fused data of e‐nose and HSI. Eventually, a comparison of models was performed by calculating statistical criteria.

#### 2.3.1 E‐nose data extraction

**FIGURE 2:**
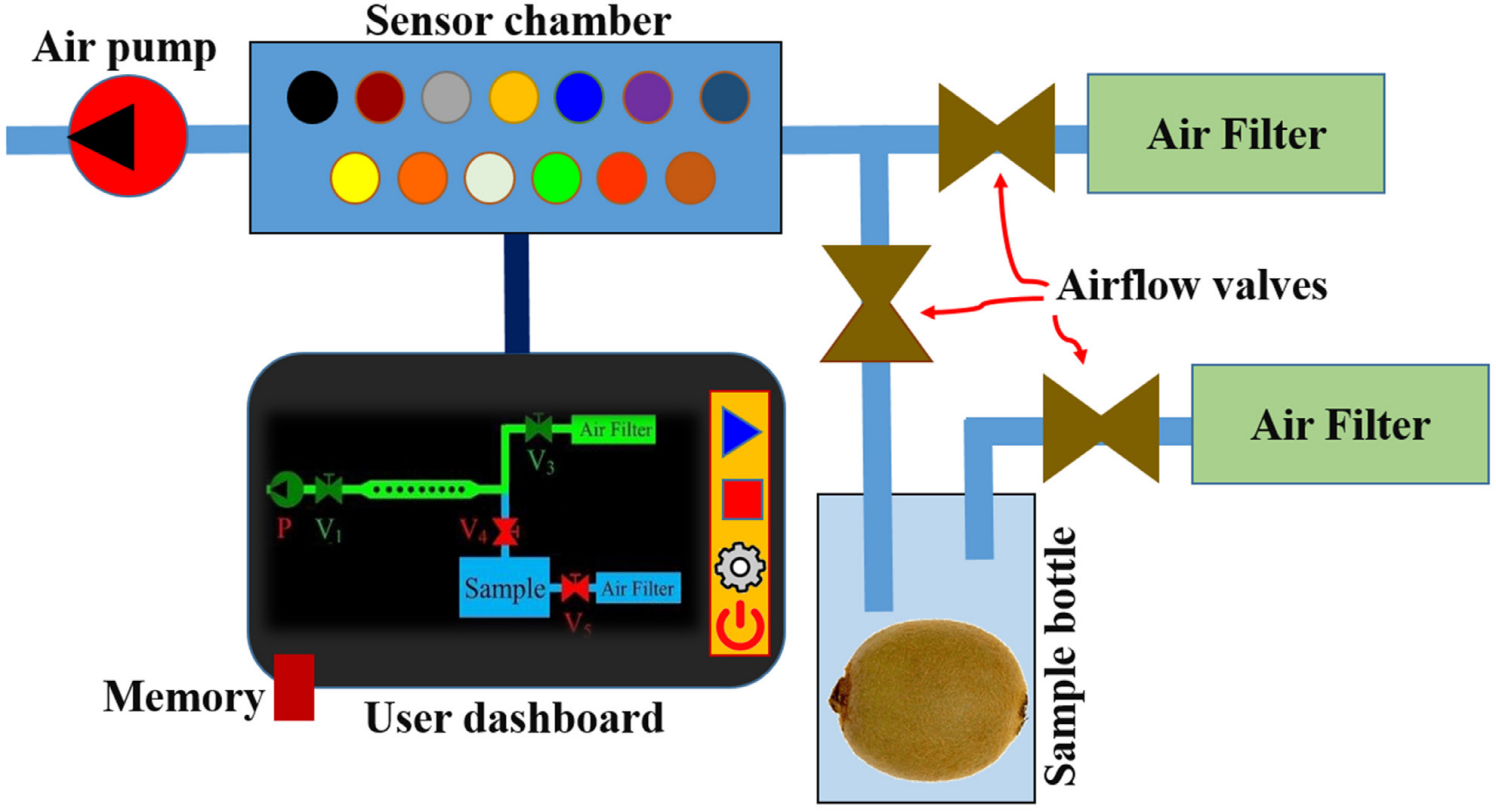
Schematic view of the e‐nose system.

In this study, an experimental e‐nose system consisting of eight MQ sensors, including MQ2, MQ3, MQ4, MQ5, MQ7, MQ8, MQ9, and MQ135 (Hanwei Electronics Co., Ltd.), and five TGS sensors, including TGS813, TGS822, TGS2610, TGS2602, and TGS2620 (Figaro Electronic Co., Ltd.), were used to obtain kiwifruits aroma information. The system also comprised a sensor chamber, a sample bottle, active carbon air filters, air transmission hoses, solenoid valves, an electronic board, an air pump, a graphical user dashboard interface, and a data storage memory. A schematic view of the e‐nose system is presented in Figure 2.

To saturate the sample container with the fruit aroma, the kiwifruit samples were kept inside the container for 15 min before data acquisition. Then the signal recording operation started, which consisted of three stages with a total duration of 310 s. The first stage was 210 s of cleaning the sensor chamber with filtered air. The second stage was a 40‐s time of passing sample headspace odor into the sensor chamber. This caused the feedback of each sensor in the sensor chamber according to its reaction to the aroma gas contents. The third stage was a 60‐s step of passing the filtered air again into the sensor chamber to clean the remaining scent from the sensors' surroundings. The sensors' feedback signals were recorded in a memory, transferred into the computer, and loaded in the MATLAB programming software (Version 2021a, the MathWorks, USA) for further analysis. Before feature extraction, the fractional method was applied for data baseline correction and normalization using equation (1), where the $X_{s}\left(0\right)$, $X_{s}\left(t\right)$, and $Y_{s}\left(t\right)$ are the baseline response, the raw response, and the manipulated response, respectively (Aghilinategh et al., 2020).

(1) $$Y_{s}\left(t\right)=\frac{X_{s}\left(t\right)-X_{s}\left(0\right)}{X_{s}\left(0\right)}$$

**FIGURE 3:**
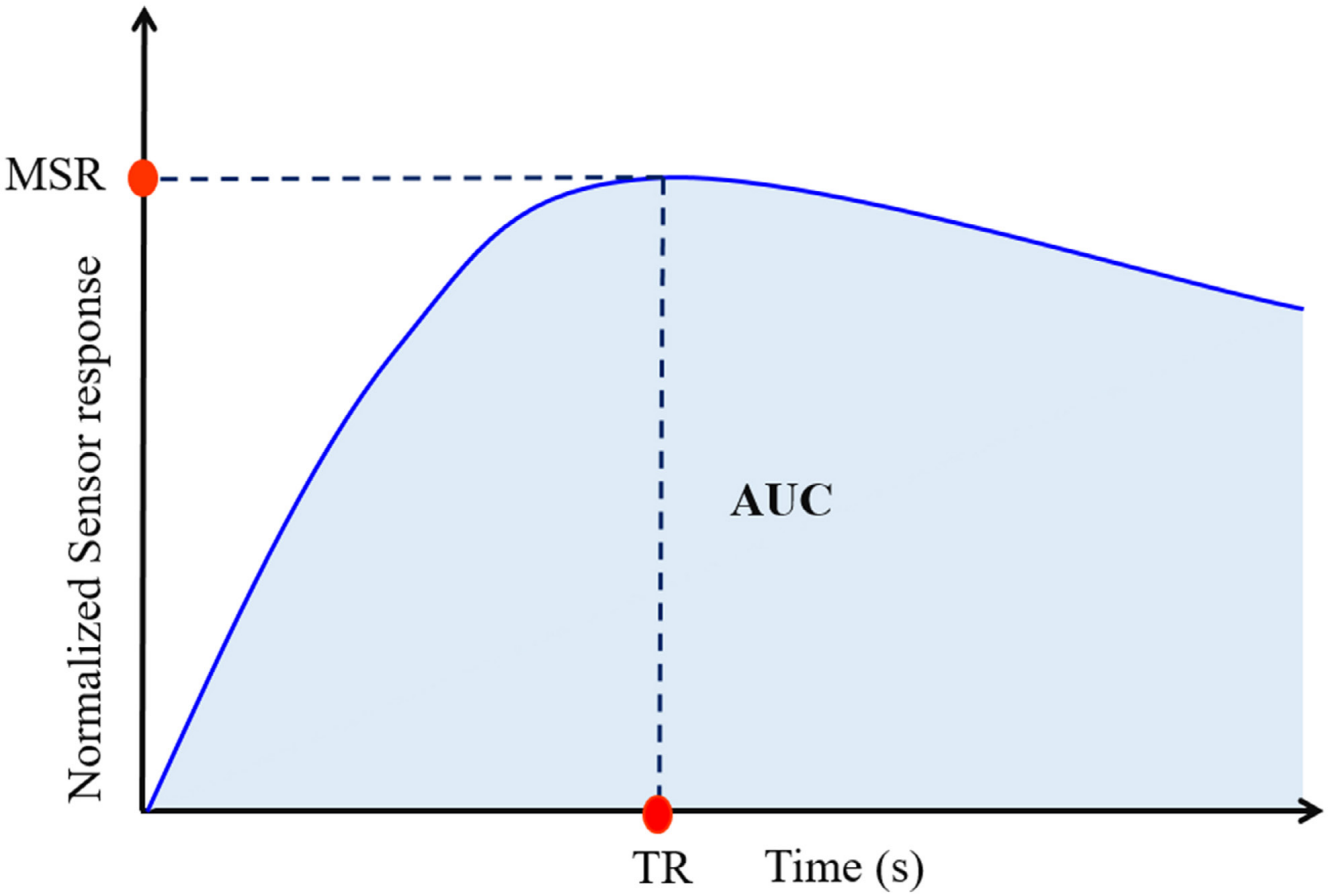
Graphical indication of the e‐nose extracted features.

After baseline correction, the data of the headspace collection phase between 211 and 250 s were used to determine the e‐nose features. Three features were calculated from the manipulated sensor responses, including maximum sensor response (MSR), area under the curve (AUC), and the time to reach MSR, also called impregnation time (Tim). These features are schematically shown in Figure 3 and well described in previous literature (Gancarz et al., 2021; Li, Ren, et al., 2019; Sanaeifar et al., 2021). A total number of 39 e‐nose features (13 sensors × 3 features) were extracted for each sample in this case.

#### 2.3.2 Hyperspectral image acquisition and feature extraction

**FIGURE 4:**
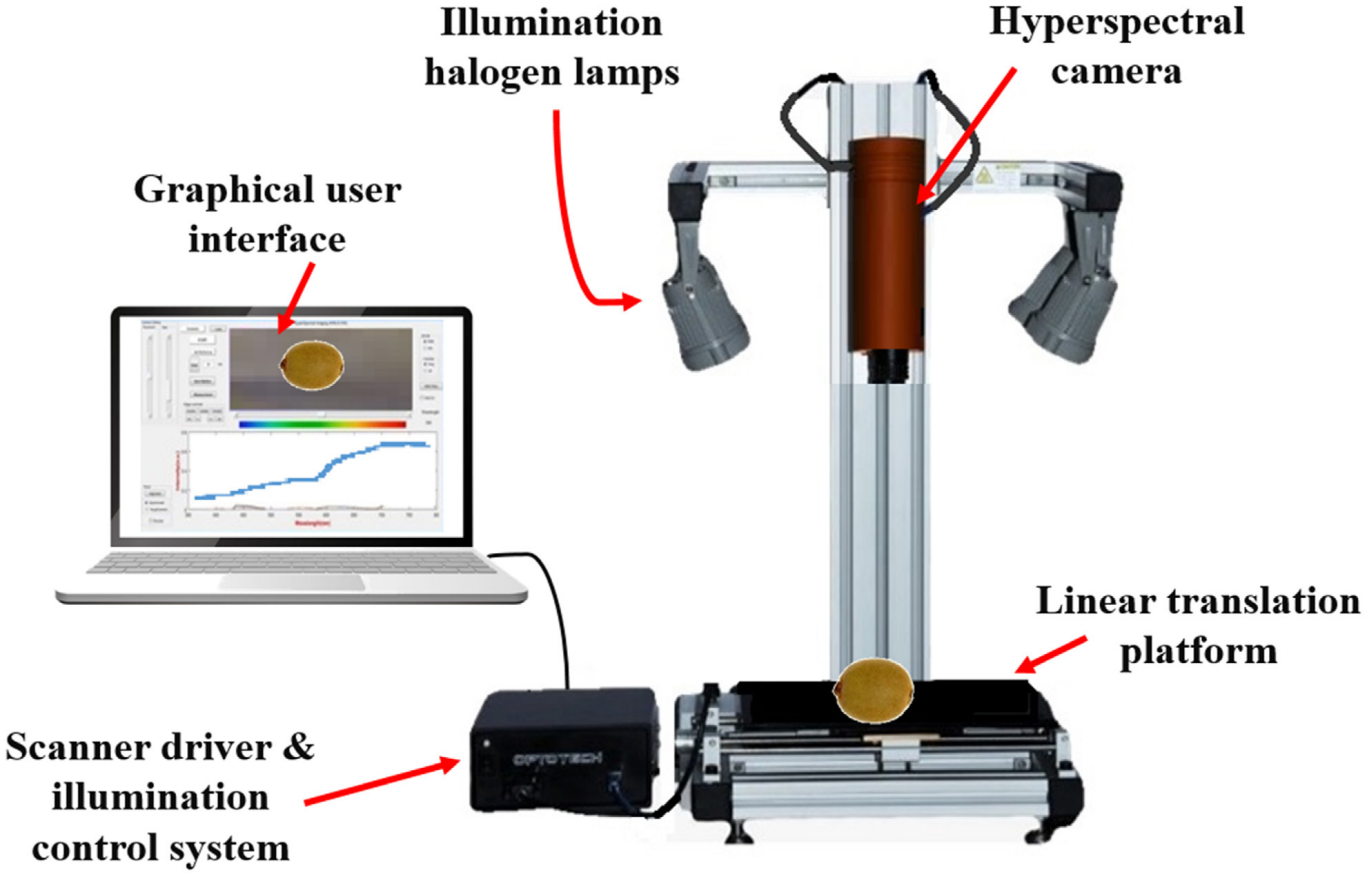
Parts of HSI system.

An HSI system (HYSPIM, Model HS_Vis‐NIR‐15fps, Iran) comprising a hyperspectral camera, a linear translation platform, illumination halogen lamps, a scanner driver, an illumination control system, and a processing software with a graphical user interface (Figure 4) was used for acquiring hyperspectral datacubes from kiwifruit samples. The vertical distance of the camera from the sample was 25 cm, and the linear speed of the platform was adjusted 3 mm/s. The images were captured with an exposure time of 0.3 s in the wavelength range of 400–950 nm with 568 wavelengths. The hypercubes were dark and white calibrated. The dark reference image was captured in a dark room with turned‐off illumination lights and a closed camera lens with a black cap. A company‐provided white tile with a reflectance of more than 99% was employed to take a white reference image. The calibration was performed using equation (2), in which the $I_{c}$, $I_{r}$, $I_{d}$, and $I_{w}$ are the calibrated hyperspectral image, raw hyperspectral image, dark reference, and white reference, respectively (Liu et al., 2018).

(2) $$I_{c}=\frac{I_{r}-I_{d}}{I_{w}-I_{d}}$$

Initial visual evaluations showed that the noise‐to‐signal ratio of the image is very high in the ranges of 400–450 nm and 900–950 nm. Therefore, the images of these spectral ranges were excluded from further calculations, and the spectral range of 450–900 nm was used for machine learning operations.

To get spectral reflectance data from the images, the hypercubes were loaded in MATLAB software. Four 5 × 5 blocks were randomly selected from different regions of the fruit surface to eliminate the effect of spectra collection position. The overall average spectral value of all pixels in four blocks of each fruit was selected as the representative reflectance spectrum of that fruit.

Preprocessing is a necessary step in spectral analysis to abolish the unwanted variations in the spectra due to noise, scattering issues, and baseline shift problems, to promote the performance of predictive models (Chia et al., 2013; Nagel‐Held et al., 2022). In this study, three different spectra preprocessing algorithms were applied to the raw data, namely moving window smoothing (MWS), first‐order Savitzky–Golay derivative (SG1D), and standard normal variate (SNV). MWS is a typical spectrum pretreatment method that can reduce local noises (Kang et al., 2013). SNV is usually used in spectra preprocessing to eliminate the impact of scattering from a spectrum (Witteveen et al., 2022). The SG1D algorithm can eradicate the interfering noise of the background and discriminate overlapping peaks to increase the resolution (Cheng et al., 2022; Wang et al., 2018). The second‐order polynomial was used for SG1D in this study. The window size was five points in this study for all pretreatment algorithms.

Due to the large number of spectral wavelengths, and probable irrelevant data, redundancy, and intercorrelations, the CARS algorithm was employed to extract the significant wavelengths. CARS is one of the most commonly used wavelength selection techniques in chemometrics to select the optimal wavelengths (Li et al., 2009; Yu et al., 2021). In CARS technique, the significant wavelengths are determined based on the Monte Carlo resampling method and the high *R* ^2^ values of the PLS model (Wang et al., 2022).

### 2.4 Model establishment

The e‐nose and hyperspectral data were used separately and in combination to classify the kiwifruit samples based on storage time and to predict the changes in quality attributes during the ripening process. SVM and PLSDA models were used for classification and support vector regression (SVR) and partial least squares regression (PLSR) models were used for prediction purposes.

SVM is a supervised kernel‐based classification method based on learning theory that maps the data into a hyperplane to the margin between classes (Nagasubramanian et al., 2018). SVR is a modification of SVM for the regression problems. Two‐dimensional polynomial SVR was used and the optimal values of penalty coefficient (*c*) and kernel function parameter (*γ*) were obtained by grid search method among different numbers of *c* (0.01, 0.1, 1, 10, and 100) and *γ* (0.01, 0.1, 1, 10, and 100). The best combination of *c* and *γ* was used based on the smallest validation RMSE.

PLSR is a multivariate linear regression model that seeks the optimal number of linear combinations of independent variables, called latent variables (LV) or factors, that maximizes the covariance between independent and dependent variables (Caetano et al., 2007). The smaller number of latent variables is important for the model stability (Wu et al., 2012). The optimal number of latent variables in PLS models was determined based on the lowest obtained RMSE value of the 10‐fold cross‐validation during model calibration.

PLSDA classifier is an adaptation of PLS for discriminating different classes that are defined as dummy binary vectors (Roussel et al., 2014).

The data were randomly divided into two subsets for model calibration (70%) and evaluation (30%). The 10‐fold cross‐validation algorithm was employed during training. The best models were chosen based on the lowest value of RMSE of validation.

### 2.5 Model performance evaluation

Statistical criteria were calculated to compare different developed models. The RMSE and *R* ^2^ measures were used to select the best prediction models, while the RMSE and accuracy values were used to determine the most desired classifiers. The lower the RMSE value and the higher the *R* ^2^ and accuracy values, the more appropriate the model. These statistics were calculated using Equations (3), (4), (5). In these formulas, the $y_{p,i}$, $y_{o,i}$, and $y_{o,\mathrm{ave}}$ are the *i*th predicted, the *i*th observed, and the average observed data, respectively. The letter *N* stands for the total number of samples (Yan et al., 2021). Eventually, the tp, fp, tn, and fn values are the true positive, true negative, false positive, and false negative values extracted from the confusion matrix of the classifier (Zhang et al., 2023).

(3) $$R^{2}=1-\left[\frac{\sum_{i=1}^{N}{\left(y_{o,i}-y_{p,i}\right)}^{2}}{\sum_{i=1}^{N}{\left(y_{o,i}-y_{o,\mathrm{ave}}\;\right)}^{2}}\right]$$

(4) $$\text{RMSE}={\left[\frac{1}{N}\sum_{i=1}^{N}{\left(y_{o,i}-y_{p,i}\right)}^{2}\right]}^{0.5}$$

(5) $$\text{Accuracy}=\frac{\mathrm{tn}+\mathrm{tn}}{\mathrm{tp}+\mathrm{fp}+\mathrm{tn}+\mathrm{fn}}\times 100$$

## 3 RESULTS AND DISCUSSION

The e‐nose and hyperspectral data were used in individual and combined modes to classify kiwifruits and to predict the changes in their physiochemical characteristics during the postharvest ripening process. Thereupon, the effect of ripening process on the physiochemical attributes of kiwifruit is presented in section 3.1. The results of the e‐nose system in monitoring the ripening process of kiwifruit are available in section 3.2. In section 3.3, the results of the HSI system are reported. Eventually, in section 3.4, the results of the data fusion‐based models for tracking the ripening procedure and quality attributes of green Hayward kiwifruit are discussed.

### 3.1 Kiwifruit quality indices

**FIGURE 5:**
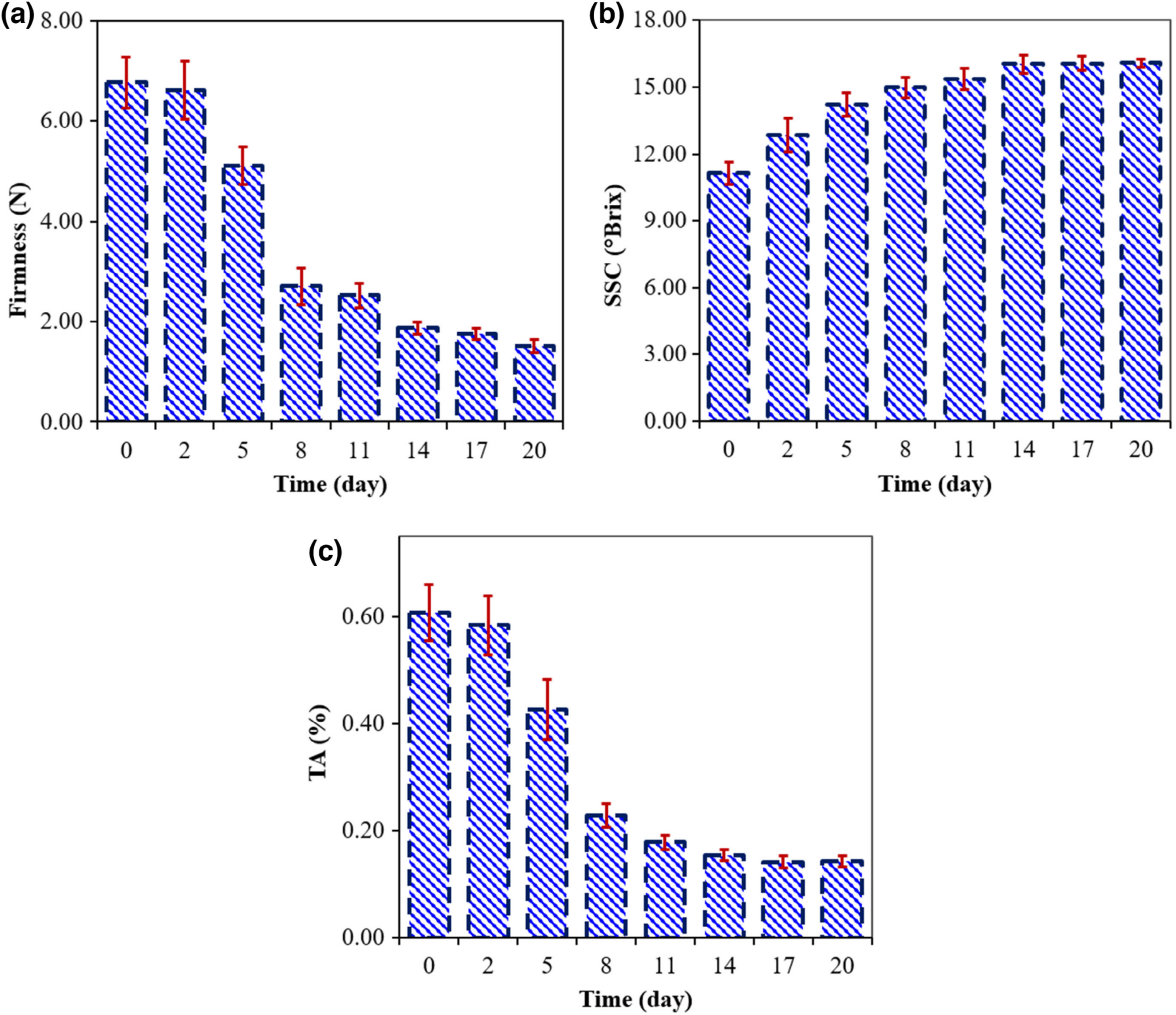
Variations of the firmness (a), SSC (b), and TA (c) values of the kiwifruit samples during the experiment.

The analysis of variance (ANOVA) results of the effect of time on the physiochemical characteristics of kiwifruits are shown in Table 1. This table shows that time has a significant impact (*p* < .01) on all three attributes studied, which indicates that the ripening process has a considerable effect on the quality characteristics of kiwifruit. The variations in quality characteristics during postharvest storage are shown graphically in Figure 5. Dissimilar letters above the bars state significant differences between average values based on Tukey's HSD post hoc analysis (*p* < .05). According to Figure 5, the fruit quality attributes have changed over time. The most significant variations were between the second and eighth days. Meanwhile, almost no significant changes observed during days 14–20.

**TABLE 1:** Results of ANOVA performed on the firmness, SSC, and TA of kiwifruit samples versus time treatment.

Attribute	Source of variation	Sum of squares	df	Mean square	*F*	*p*
Firmness	Time (day)	470.862	7	67.266	1590.270	.000
	Error	4.737	112	0.042		
	Total	475.599	119			
SSC	Time (day)	341.075	7	48.725	546.374	.000
	Error	9.988	112	0.089		
	Total	351.063	119			
TA	Time (day)	4.016	7	0.574	2172.266	.000
	Error	0.030	112	0.000		
	Total	4.046	119			

The ripening process degrades the xyloglucans and polyuronide substances in the fruit tissue. This leads to the collapse of cell wall structure, which in turn decreases the flesh firmness of the fruit (Wakabayashi, 2000). The increase in the kiwifruit SSC during the ripening process, as seen in Figure 5b, is mainly due to the hydrolyzing of starch to simple sugars such as fructose and glucose (Macrae et al., 1989; Shahkoomahally & Ramezanian, 2015). As observed in Figure 5c, the TA value of the kiwifruit decreased during the storage. This variation is due to the metabolism of glucose and the tricarboxylic acid cycle (Ding et al., 2013). Similar variation trends were reported in the SSC, TA, and firmness of Hayward kiwifruit in recent studies (Asiche et al., 2017; Chai et al., 2021; Huang et al., 2019).

### 3.2 Results of e‐nose

#### 3.2.1 Study on the e‐nose features

**FIGURE 6:**
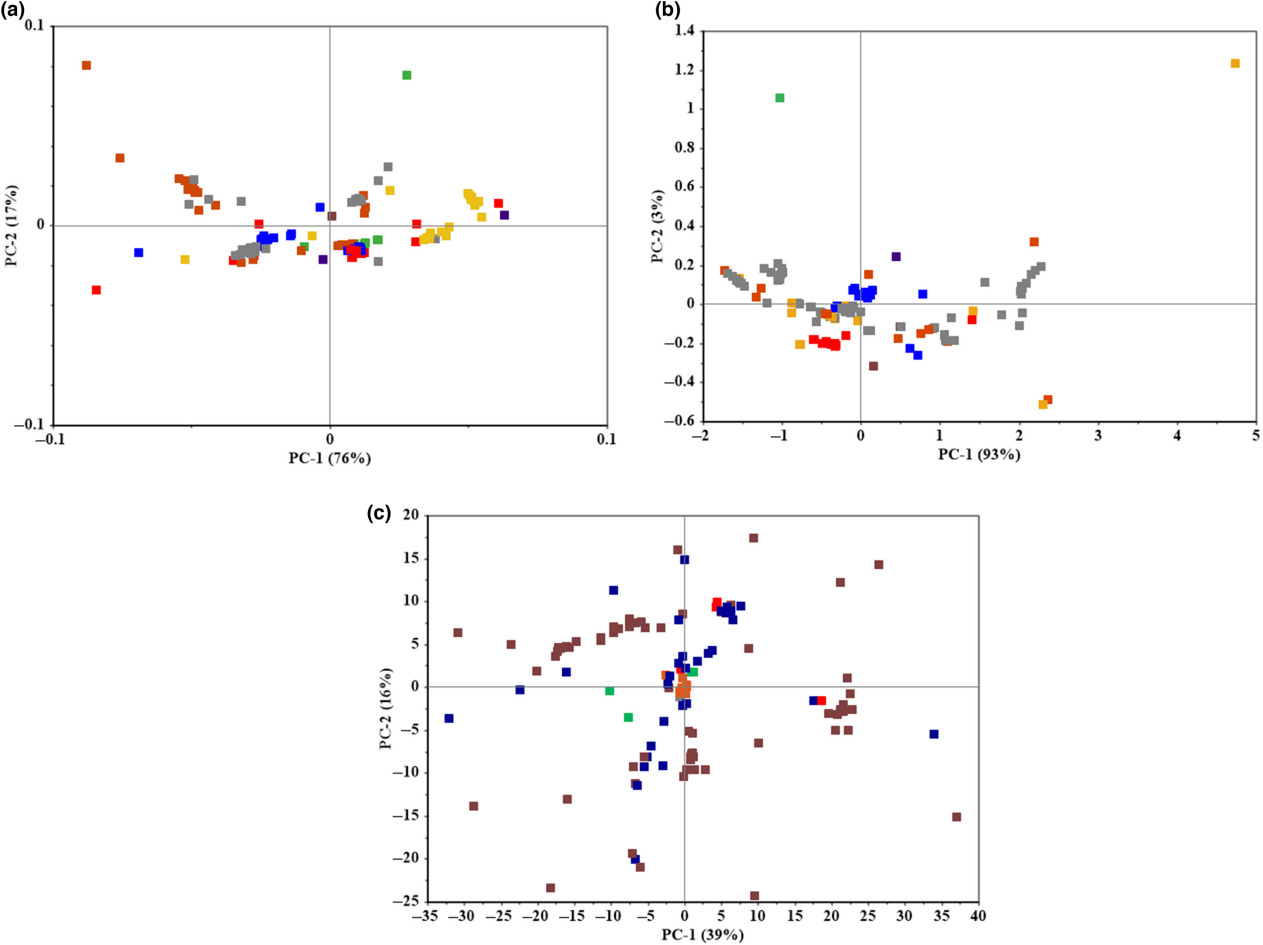
Score plots of the PCA of the e‐nose features: (a) MSR, (b) AUC, and (c) TR (day 0 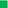, day 2 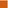, day 5 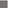, day 8 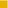, day 11 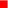, day 14 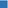, day 17 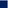, day 20 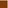).

Figure 6 shows the score plots generated by PCA on the e‐nose features. Figure 6a depicts that the first two PCs of the MSR feature were able to cover 93% (PC1 = 76%, PC2 = 17%) of the variance of data. The variance coverages of the first two PCs were 96% (PC1 = 93%, PC2 = 3%) and 55% (PC1 = 39%, PC2 = 16%) for AUC (Figure 6b) and TR (Figure 6c) features, respectively. Therefore, the first two PCs extracted from MSR and AUC features are good representatives of the information contained in these features. In addition, it is observed that the PC1 and PC2 vectors cannot clearly distinguish between different classes. This shows the importance of selecting the optimal number of PCs, or latent variables, to reach accurate results in PC‐based models such as PLS.

**FIGURE 7:**
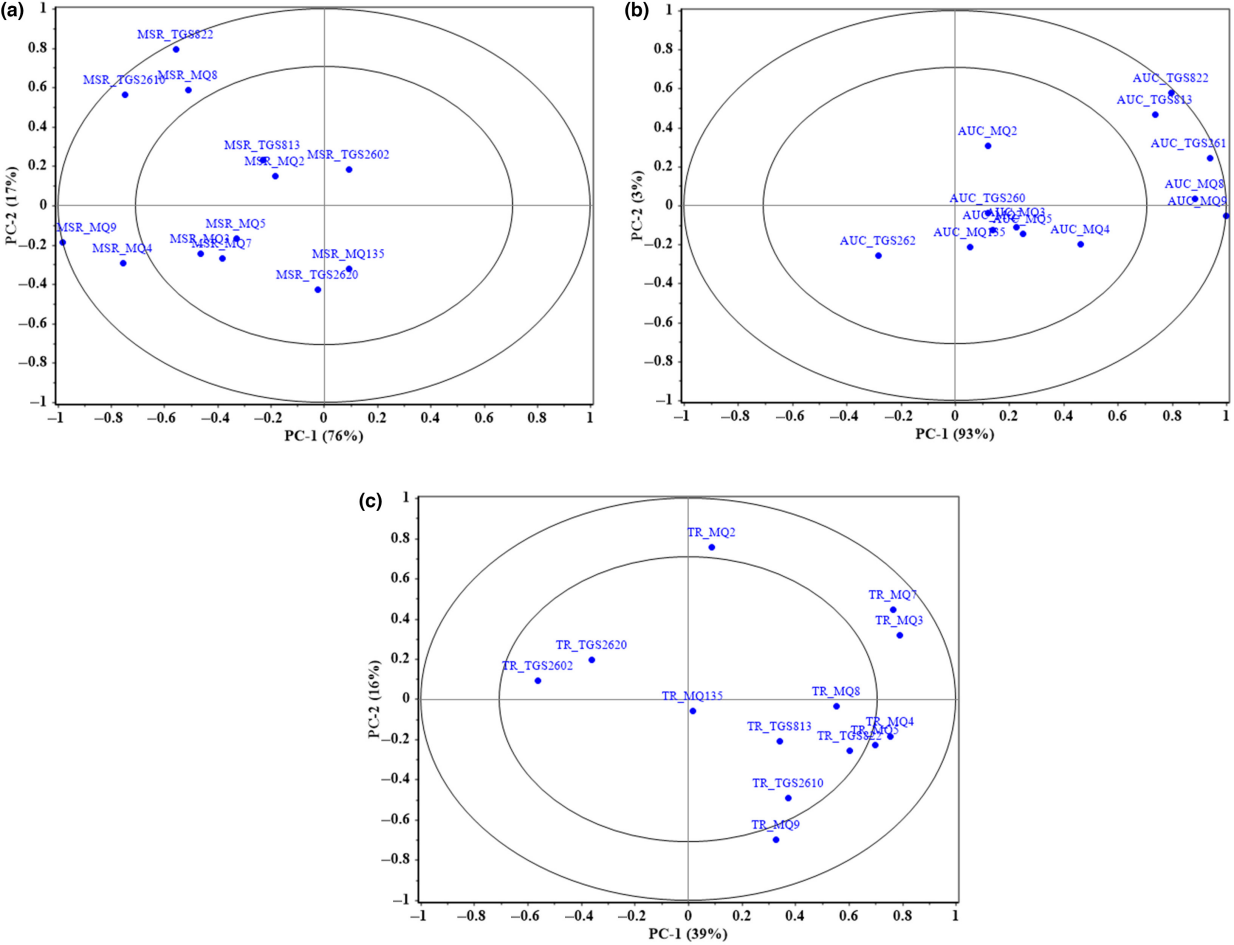
Loading plots of the PCA of the e‐nose features: (a) MSR, (b) AUC, and (c) TR.

**FIGURE 8:**
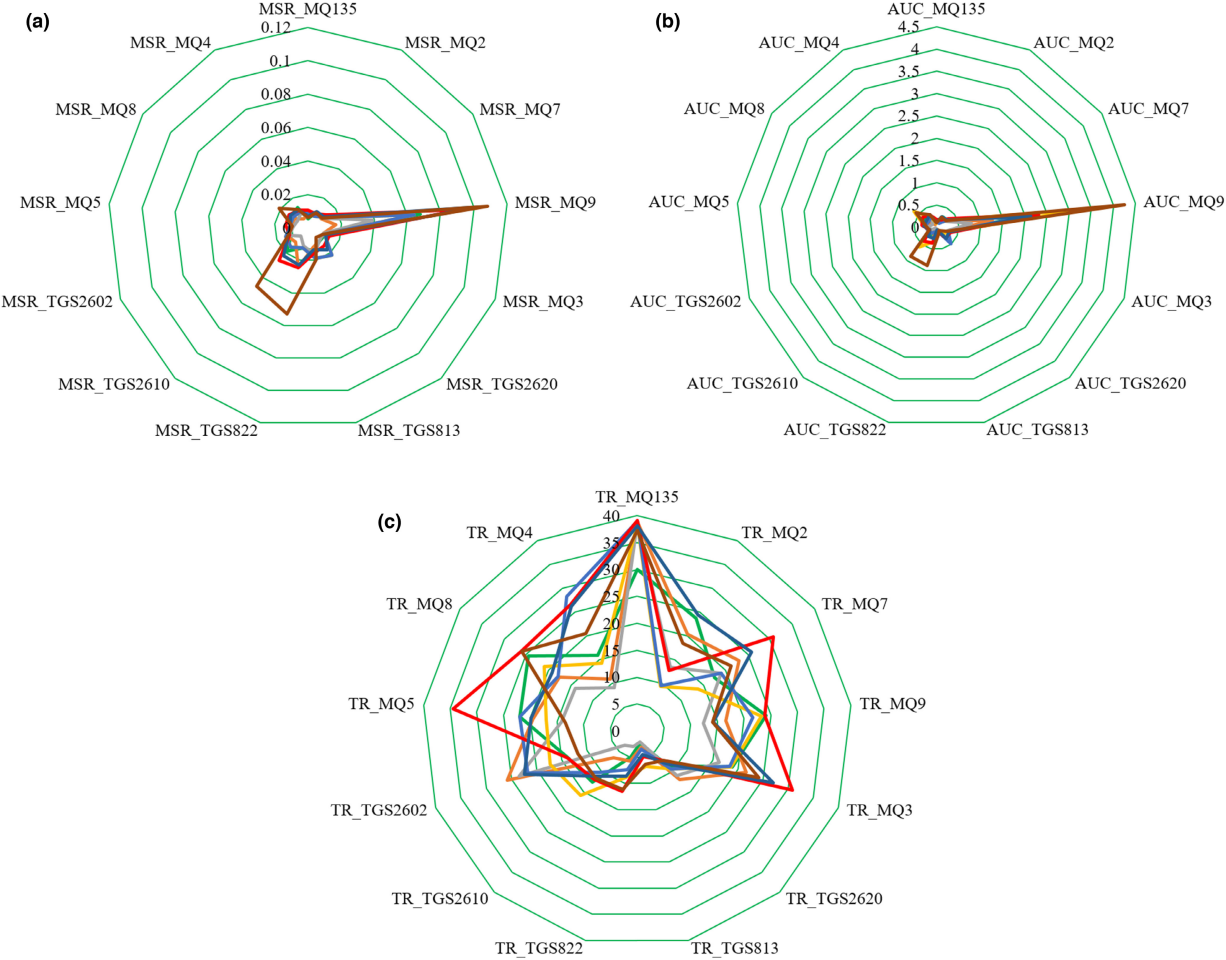
Radar plots of e‐nose features: (a) MSR, (b) AUC, and (c) TR (day 0 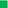, day 2 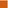, day 5 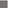, day 8 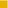, day 11 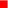, day 14 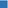, day 17 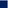, day 20 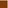).

PCA loading plots (Figure 7) were used to investigate the correlations between the e‐nose features and the clusters of kiwifruit samples. The higher loading values indicate the higher contribution of the element for determining the target item. Figure 7a shows that the MSR values of TGS822, TGS2610, and MQ9 sensors, which are closest to the circumference of the outer circle, have the most critical effect on the discrimination of kiwifruit during ripening. The AUC features of these three sensors also have the highest contributions to fruit ripening detection (Figure 7b). Figure 8 helps to explain this point better. The radar plots of the extracted features from sensor responses are available in Figure 8. Different days are depicted in different colors. It is seen that the feature averages have dissimilar values for different days. From Figure 8a,b, it is obvious that the MSR and AUC values of the TGS822, TGS2610, and MQ9 sensors have increased with the increase of the storage duration. The ripening process increases the emission of ripeness‐dependent VOCs, especially hydrocarbon derivative groups (Brezmes et al., 2000), which increases the reaction of these three hydrocarbon‐sensitive sensors. For example, the concentration of ethyl acetate, which is a highly aromatic hydrocarbon, rises significantly as the kiwifruit starts to soften due to ripening (Yen & Yao, 2021).

A different behavior was observed in TR feature, where the MQ2, MQ3, and M7 sensors were the most important ones (Figure 7c). On the other side, Figure 8c shows that TR values do not offer an evident tendency in most of the sensors. The results of this section show that removing a specific type of feature or a particular sensor may decrease the accuracy of the algorithms. Therefore, all data extracted from sensors were included to create classification and prediction models.

#### 3.2.2 Results of e‐nose‐based models

Table 2 shows the results of the e‐nose data‐based developed models for classifying the kiwifruit samples and predicting the quality characteristics of the samples during the ripening. From this table, it is apparent that the polynomial SVM model with a penalty value of 0.01 and *γ* of 10 was the most optimal classifier with the validation RMSE (RMSEV) of 0.112. The validation *R* ^2^ (*R* ^2^V) of this structure was 0.916. This classifier could discriminate the kiwifruit samples according to their storage time with accuracies of 98.8% and 90.5% in the calibration and validation stages, respectively. The model was also evaluated on separate test data, and an accuracy of 91.67% was obtained.

**TABLE 2:** Performance criteria of the classification and prediction models for monitoring the kiwifruit ripeness process using e‐nose data.

Duty	Model	Optimal parameters	*R* ^2^C	RMSEC	*R* ^2^V	RMSEV
Classification	PLSDA	LV = 12	0.930	0.086	0.869	0.118
	SVM	*c* = 0.01, *γ* = 10	0.978	0.069	0.916	0.108
Firmness prediction	PLSR	LV = 16	0.959	0.398	0.820	0.830
	SVR	*c* = 1, *γ* = 100	0.990	0.245	0.932	0.544
SSC prediction	PLSR	LV = 17	0.969	0.297	0.853	0.648
	SVR	*c* = 100, *γ* = 0.1	0.988	0.211	0.949	0.340
TA prediction	PLSR	LV = 16	0.967	0.033	0.790	0.084
	SVR	*c* = 10, *γ* = 1	0.991	0.021	0.947	0.045

The e‐nose features were fed into PLSR and SVR models to predict the variations of kiwifruit firmness, SSC, and TA attributes during postharvest storage. It was observed that the SVR algorithm was a more accurate predictor of quality attributes than the PLSR algorithm. The calibration *R* ^2^ (*R* ^2^C) and RMSE (RMSEC) criteria of the SVR model for firmness prediction based on e‐nose data were 0.990 and 0.245, respectively. This model obtained the *R* ^2^V of 0.932 and RMSEV of 0.544 during 10‐fold cross‐validation. The *R* ^2^ and RMSE values of the e‐nose‐based SVR model for firmness prediction in the test dataset were obtained as 0.901 and 0.714, respectively. These accuracy values prove the capability to use VOC changes in predicting fruit firmness during storage. The ripening process changes several VOC compositions such as terpene and aromatic alcohols, which are precursors of fruits' pleasant aromas. These compounds are bound by glycosides, and are only released during ripening (Lebrun et al., 2008). Other VOCs, such as esters, lactones, aldehydes, and ketones, are produced during the ripening process of kiwifruit (Kim et al., 2023). It was reported that the kiwifruits with different firmnesses during the ripening have significantly different contents of volatile organic compounds that can be used to discriminate the samples with different degrees of ripeness (Chai et al., 2022).

According to Table 2, in the case of SSC prediction, the SVR model gained the *R* ^2^V and RMSEV values of 0.949 and 0.340, respectively. This model achieved the *R* ^2^ of 0.925 and RMSE of 0.526 for SSC prediction in the e‐nose test dataset. Higher accuracy and superiority of the SVR algorithm compared to PLSR were also reported in a related study conducted by Du, Wang, et al. (2019), where the *R* ^2^ values of the SVR model on the test dataset were 0.929 and 0.914 for the prediction of kiwifruit firmness and SSC measures, respectively.

Eventually, the SVR model resulted in the *R* ^2^V and RMSEV of 0.947 and 0.045, respectively, for kiwifruit TA estimation during the ripening process. The *R* ^2^ and RMSE values of the e‐nose‐based SVR model for kiwifruit TA predicting in the test data were 0.939 and 0.047, respectively.

These obtained performance criteria confirm the appropriate potential of the e‐nose system for nondestructive monitoring of the kiwifruit postharvest ripening. According to literature, a characteristic event during the ripening of kiwifruit, besides its softening, acidity reduction, and starch‐to‐sugar conversion, is the aroma development (Asiche et al., 2017; Liu et al., 2021). The concentration of ripeness‐dependent VOCs is positively correlated with SSC, and negatively correlated with flesh firmness (Sanchez et al., 2012). Therefore, monitoring of aroma variations gives important information about the ripening stage and physiochemical indices of kiwifruit.

These results conform to those reported about the successful prediction of firmness, SSC, and TA values of red‐fleshed kiwifruit during the ripening process using e‐nose data, with the outperformance of the SVR model compared with PLSR (Du, Xu, et al., 2019).

Different accuracies of the e‐nose system in fruit ripening monitoring have been reported. For example, Aghilinategh et al. (2020) used a laboratory fabricated e‐nose machine with 10 metal oxide semiconductor (MOS) gas sensors to detect the ripeness level of blackberry and whiteberry. The obtained accuracies of the ANN model for the classification of samples into five classes were 88.3% and 100% for whiteberry and blackberry, respectively. An experimental e‐nose system was employed by Sanaeifar et al. (2016) to monitor the quality attributes of bananas during the shelf‐life storage period. The variations of firmness, total soluble solid (TSS), pH, and TA values of banana fruits during the experiment were predicted by the SVR model with the determination coefficients (*R* ^2^) of 0.8852, 0.9608, 0.7607, and 0.7033, respectively.

### 3.3 Results of HSI

#### 3.3.1 Results of CARS wavelength selection

**FIGURE 9:**
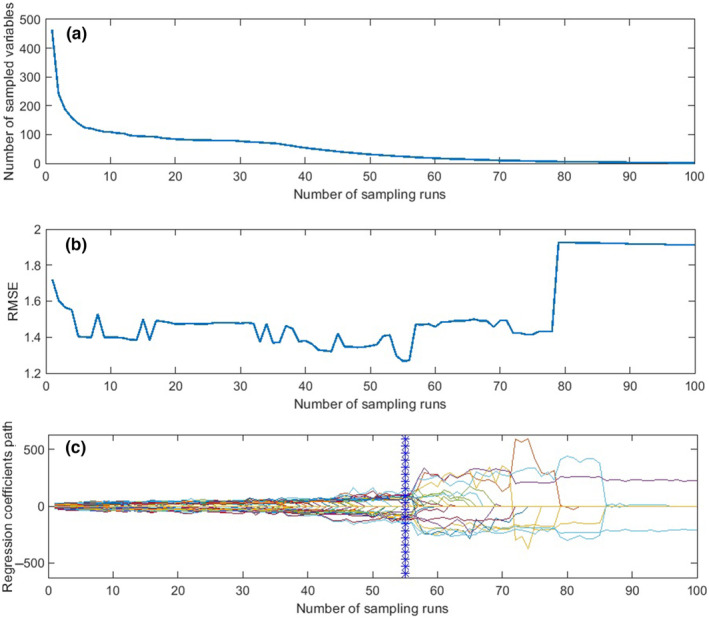
Graphical result of CARS on the MWS spectra for kiwifruit firmness prediction, displaying the number of sampled wavelengths (a), the RMSE (b), and the regression coefficients (c) at different sampling runs.

Table 3 shows the number of CARS‐selected wavelengths for classification and prediction objectives in different pretreatment methods. As an example, for firmness prediction, 54 wavelengths from the total 465 wavelengths (about 11.61%) were selected by applying CARS algorithm on MWS‐treated spectra. The results of the CARS algorithm on MWS processed data for predicting fruit firmness is presented in Figure 9. Figure 9a displays that the number of selected wavelengths decreased sharply in the early runs due to the exponential decay function (EDF) used in the wavelength selection algorithm (Yun et al., 2015). The RMSE measure also decreases sharply during this stage (Figure 9b). In the next step, the downward trend of the sampled variables becomes slower, along with a slighter decrease in RMSE. This stage is called refined selection stage. Eventually, the RMSE increases again at the sampling run 57, highlighted by an asterisk line in Figure 9c. The number of remained wavelengths at this run was 54. With a same procedure, 41, 72, and 45 wavelengths were selected by CARS algorithm from MWS‐preprocessed spectra for kiwifruit ripeness classification, SSC prediction, and TA prediction, respectively.

**TABLE 3:** The number of CARS‐selected wavelengths for classification and prediction purposes.

Spectra	Classification	Firmness prediction	SSC prediction	TA prediction
MWS	41	56	72	45
SG1D	37	67	51	51
SNV	34	64	39	44

#### 3.3.2 Results of HSI‐based models

The CARS‐selected features were used to establish models for noninvasive monitoring of kiwifruit ripening process. The resulting criteria of developed models are provided in Table 4. Comparing Table 4 with Table 2 shows that the hyperspectral data‐based models were less accurate than e‐nose‐based models. The physicochemical changes that occur during the fruit ripening process have a greater effect on the odor emitted from the fruit than the appearance of the fruit. Therefore, the odor‐extracted features are better descriptors of fruit ripening than the spectral reflectance. From Table 4, the SG1D‐based models were the most accurate algorithms among different treatment algorithms. In addition, this table shows that the PLSDA models were more accurate classifiers than the SVM models for discriminating the kiwifruits based on storage time. Consequently, the most accurate spectral‐based kiwifruit ripeness classifier was the SG1D‐PLSDA structure, with the *R* ^2^C and RMSEC measures of 0.928 and 0.099, and *R* ^2^V and RMSEV of 0.881 and 0.115, respectively. This model obtained the *R* ^2^T and RMSET of 0.886 and 0.113, with a classification accuracy of 83.33% in test data.

**TABLE 4:** Performance criteria of the classification and prediction models for monitoring the kiwifruit ripeness process using hyperspectral data.

Duty	Model	Spectra pretreatment	Optimal parameters	*R* ^2^C	RMSEC	*R* ^2^V	RMSEV
Classification	PLSDA	MWS	LV = 12	0.921	0.105	0.853	0.120
		SG1D	LV = 14	0.928	0.099	0.881	0.115
		SNV	LV = 11	0.919	0.107	0.874	0.117
	SVM	MWS	*c* = 100, *γ* = 10	0.894	0.109	0.868	0.119
		SG1D	*c* = 100, *γ* = 10	0.911	0.110	0.869	0.119
		SNV	*c* = 10, *γ* = 10	0.906	0.113	0.848	0.125
Firmness prediction	PLSR	MWS	LV = 9	0.903	0.609	0.845	0.774
		SG1D	LV = 10	0.980	0.274	0.926	0.539
		SNV	LV = 8	0.926	0.532	0.864	0.733
	SVR	MWS	*c* = 10, *γ* = 1	0.955	0.417	0.722	1.093
		SG1D	*c* = 100, *γ* = 100	0.986	0.250	0.780	0.918
		SNV	*c* = 0.1, *γ* = 100	0.982	0.266	0.753	1.021
SSC prediction	PLSR	MWS	LV = 9	0.886	0.565	0.831	0.696
		SG1D	LV = 12	0.972	0.278	0.910	0.501
		SNV	LV = 9	0.935	0.425	0.870	0.603
	SVR	MWS	*c* = 100, *γ* = 0.1	0.967	0.304	0.707	0.973
		SG1D	*c* = 0.01, *γ* = 100	0.982	0.233	0.848	0.653
		SNV	*c* = 1, *γ* = 1	0.980	0.243	0.756	0.936
TA prediction	PLSR	MWS	LV = 9	0.892	0.0602	0.829	0.076
		SG1D	LV = 11	0.986	0.022	0.932	0.049
		SNV	LV = 8	0.934	0.047	0.882	0.064
	SVR	MWS	*c* = 10, *γ* = 1	0.975	0.029	0.678	0.113
		SG1D	*c* = 1, *γ* = 1	0.987	0.022	0.805	0.081
		SNV	*c* = 0.01, *γ* = 100	0.985	0.022	0.699	0.106

In the prediction models, though the performance criteria of the SVR models were better than those of the corresponding PLSR models in the calibration stage, the performance of the SVR models decreased in the validation stage and gave lower criteria than the PLSR models. In this situation, models with better validation criteria are preferred to avoid the possibility of overfitting. Accordingly, the PLSR models are more satisfactory than SVRs for predicting the kiwifruit ripening process based on reflectance spectra data. The *R* ^2^V and RMSEV criteria of the SG1D‐PLSR model, as the most accurate predictor of kiwifruit quality attributes, were 0.926 and 0.539 for firmness prediction, 0.910 and 0.501 for SSC prediction, and 0.932 and 0.049 for TA prediction, respectively. Evaluation of the SG1D‐PLSR models on the separated test data resulted in the *R* ^2^T and RMSET values of 0.879 and 0.785 for firmness prediction, 0.883 and 0.636 for SSC prediction, and 0.920 and 0.055 for TA prediction, respectively. These performance statistics show that the spectral reflectance of kiwifruit skin in the visible‐near‐infrared range can be considered as a worthy representative of the postharvest ripeness status of this fruit.

Different performance ranges have been reported in the literature for monitoring the maturity of fruits using HSI. For example, the accuracy values in this research are much higher than those reported by Nilsen et al. (2021) for predicting the kiwifruit SSC and firmness using hyperspectral data, uninformative variable elimination (UVE) wavelength selection, and PLSR modeling algorithm. Meanwhile, the HSI system discriminated strawberries regarding their storage time with 100% classification accuracy (Weng et al., 2020).

The 400–1000 nm hyperspectral reflectance was employed by Chu et al. (2022) for monitoring the green banana maturity process. In their study, the raw spectral data were used for model establishments. The *R* ^2^ values of the PLSR algorithm were reported to be 0.74 and 0.68 for SSC and TA prediction, respectively. The highest accuracy of 94.35% was obtained by the PLSDA model for classifying the bananas into six maturity stages.

An HSI system with a 400–1000 nm acquisition range was also employed by Shao et al. (2020) to differentiate strawberry fruits into unripe, midripe, and ripe categories. Multiplicative scatter correction (MSC) method was employed to preprocess raw spectra, and CARS wavelength selection algorithm was used. The MSC‐CARS‐SVM model classified the fruits with an accuracy of 96.7%. In other studies, acceptable performance of HSI and chemometrics was reported for the nondestructive quality assessment of orange (Salah et al., 2022), Achacha (Nguyen & Liou, 2022), persimmon, pomegranate, loquat, and nectarine (Munera Picazo, 2021) fruits during postharvest storage.

### 3.4 Results of the data fusion approach

According to the results of the previous section, the CARS‐selected SG1D wavelengths yield the most desired results among different evaluated reflectance‐based models. Therefore, to create fused datasets, the CARS‐selected SG1D data were combined with the e‐nose features and used to develop classifying and predicting algorithms.

By comparing Table 5 with Tables 2 and 4, it is apparent that using the combination of olfactory and spectral data increased the accuracy of prediction and classification models compared to algorithms developed by means of individual e‐nose and hyperspectral systems.

**TABLE 5:** Performance criteria of the classification and prediction models for monitoring the kiwifruit ripeness process using fused hyperspectral and e‐nose data.

Duty	Model	Optimal parameters	*R* ^2^C	RMSEC	*R* ^2^V	RMSEV
Classification	PLSDA	LV = 15	0.943	0.077	0.885	0.112
	SVM	*c* = 0.01, *γ* = 1	0.981	0.066	0.919	0.106
Firmness prediction	PLSR	LV = 18	0.963	0.378	0.846	0.796
	SVR	*c* = 10, *γ* = 100	0.990	0.199	0.965	0.375
SSC prediction	PLSR	LV = 18	0.969	0.297	0.828	0.710
	SVR	*c* = 10, *γ* = 10	0.988	0.203	0.974	0.303
TA prediction	PLSR	LV = 17	0.967	0.033	0.841	0.073
	SVR	*c* = 1, *γ* = 1	0.991	0.018	0.974	0.030

**FIGURE 10:**
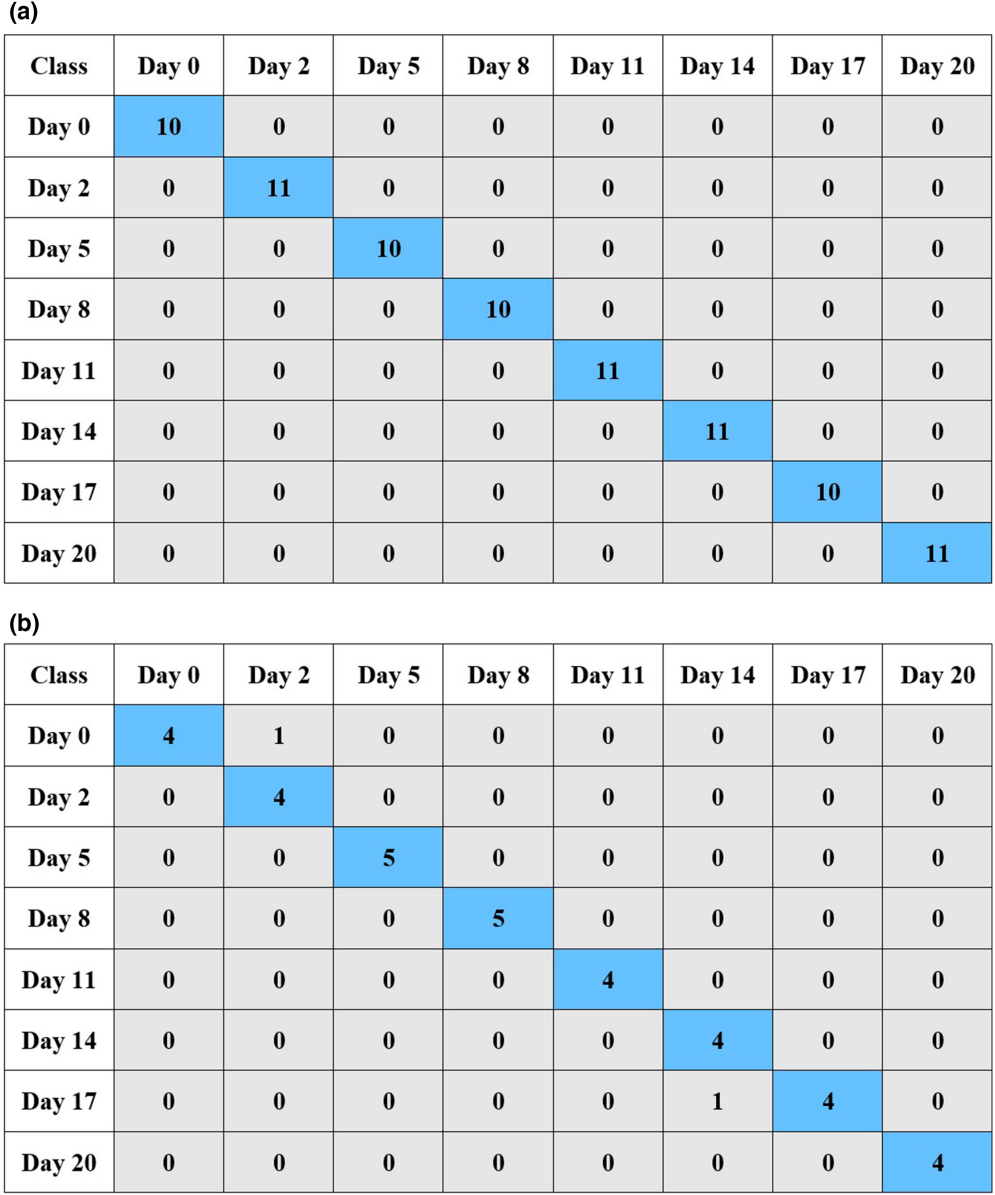
Confusion matrices of data fusion‐based SVM model for classification of kiwifruit samples based on the storage time on calibration (a) and test (b) datasets.

Referring to Table 5, the polynomial SVM classifier with the *c* value of 0.01 and *γ* of 1 was the most accurate classifier of kiwifruits using the fusion of e‐nose and spectral data, with the *R* ^2^C, RMSEC, *R* ^2^V, and RMSEV of 0.981, 0.066, 0.919, and 0.106, respectively. The classification accuracy values of this model in the calibration, cross‐validation, and test stages were obtained as 100%, 92.67%, and 94.44%, respectively. The confusion matrices of the data fusion‐based SVM classifier on calibration and test stages are available in Figure 10. It can be observed that all 84 samples of the calibration dataset were correctly classified (Figure 10a), while 2 of 36 samples were misclassified in test stage (Figure 10b).

Evaluations indicated that by using combined data, the SVR model with the *c* value of 10 and the *γ* value of 100 was able to estimate the variations of kiwifruit firmness during the ripening process with *R* ^2^C, RMSEC, *R* ^2^V, and RMSEV of 0.990, 0.199, 0.965, and 0.375, respectively. This structure predicted the fruit firmness in the test dataset with an *R* ^2^T of 0.962 and an RMSET of 0.408.

**FIGURE 11:**
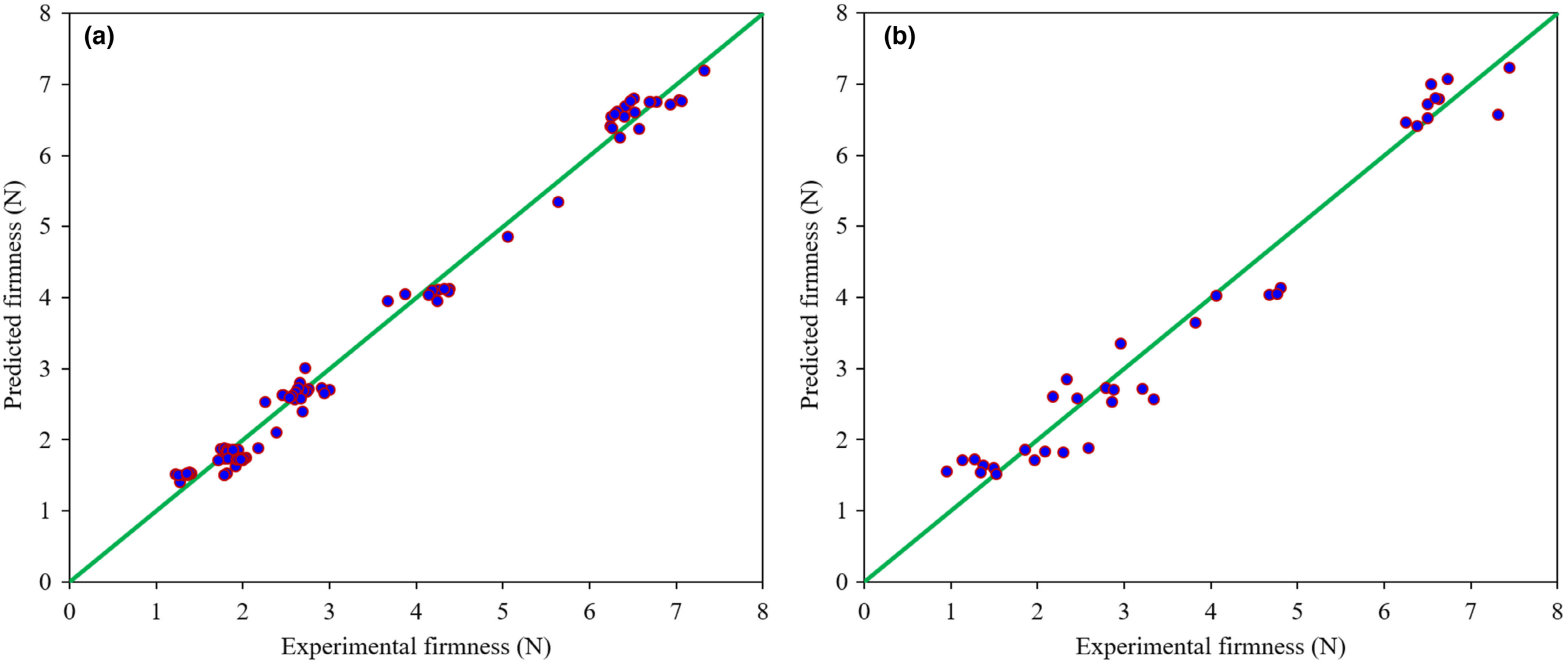
Scatter plots of the data fusion‐based SVR model for firmness prediction of kiwifruit samples during the storage on calibration (a) and test (b) datasets.

**FIGURE 12:**
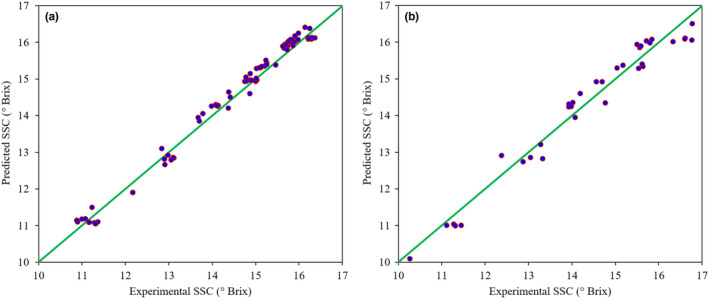
Scatter plots of the data fusion‐based SVR model for SSC prediction of kiwifruit samples during the storage on calibration (a) and test (b) datasets.

**FIGURE 13:**
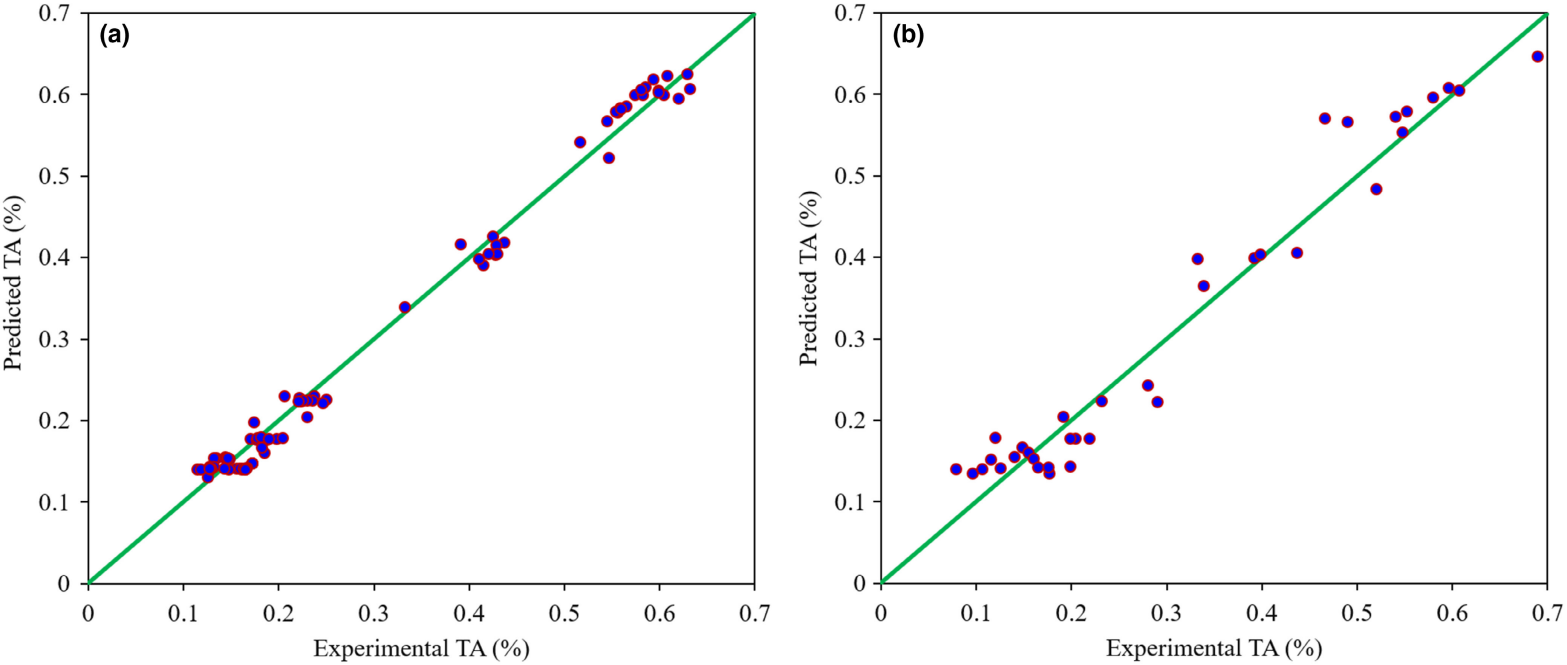
Scatter plots of the data fusion‐based SVR model for TA prediction of kiwifruit samples during the storage on calibration (a) and test (b) datasets.

In SSC modeling, the SVR model (*c* = 10, *γ* = 10) was the superior predictor with 0.974 for *R* ^2^V and 0.303 for RMSEV, leading to the *R* ^2^T of 0.964 and RMSET of 0.337 for SSC prediction in the test dataset. The SVR model also outperformed the PLSR model for monitoring the TA content of kiwifruit samples during the ripening based on the fusion of hyperspectral and e‐nose data. The *R* ^2^V and RMSEV of the SVR model in this item were 0.974 and 0.030, respectively. The *R* ^2^T and RMSET parameters of this model for TA prediction were 0.955 and 0.039, respectively. Graphical indications of the results of data fusion‐based SVR models for predicting the kiwifruit firmness, SSC, and TA attributes are provided in Figures 11, 12, 13, respectively. The close distribution of data points around the diagonal green line shows the agreement between predicted and laboratory‐measured attributes. This demonstrates the robustness of the SVR models in predicting the quality characteristics of kiwifruit during the postharvest ripening process.

The results of this research prove the applicability of data fusion to create more accurate inspection algorithms for the quality characteristics of horticultural products. Liu et al. (2019) reported that the use of the fusion of e‐nose and hyperspectral data together with the SVM algorithm increased the prediction accuracy for variations of total soluble solids (TSS) and TA contents in strawberries during the decay process, when compared to individual systems. It was also revealed in other studies that the fusion of hyperspectral and e‐nose data could improve the quality tracking performance for rice (Shi et al., 2021) and egg (Zhang et al., 2022) rather than single sensing techniques.

Considering the high‐performance values obtained in this study and regarding the advantages of both e‐nose and HSI systems, such as nondestructive evaluation, being affordable, and capability to increase the detection speed, these methods can be coupled to develop a nondestructive, low‐cost, and fast system to monitor the quality attributes of fruits during the postharvest storage, and even in sorting systems.

## 4 CONCLUSION

The feasibility of data fusion strategy, based on HSI and e‐nose systems, was investigated for nondestructive assessment of the kiwifruit postharvest ripening process. The spectral reflectance and aroma fingerprint features of kiwifruits were used for classifying the fruits and predicting their firmness, SSC, and TA values during the postharvest storage.

It was resulted that the data fusion method enhances the performance of chemometric algorithms rather than the single sensing systems. Consequently, an accuracy of 94.4% was obtained by the SVM model for classifying the fruits into eight distinct groups based on the storage days. In comparison, the classification accuracy was 83.33% by using only hyperspectral data and 91.67% by using only e‐nose data. Furthermore, the *R* ^2^ values of the data fusion‐based SVR models for predicting kiwifruit firmness, SSC, and TA measures were 0.962, 0.964, and 0.955, respectively.

In conclusion, the combination of HSI and e‐nose systems, followed by appropriate machine learning algorithms, can be effectively employed to develop a reliable nondestructive tool for monitoring the quality attributes of kiwifruit during postharvest storage.

## AUTHOR CONTRIBUTIONS

**Adel Bakhshipour:** Conceptualization (lead); data curation (lead); formal analysis (lead); funding acquisition (lead); investigation (lead); methodology (lead); project administration (lead); resources (lead); software (lead); supervision (lead); validation (lead); visualization (lead); writing – original draft (lead); writing – review and editing (lead).

## CONFLICT OF INTEREST STATEMENT

The author declares no conflict of interest.

## Data Availability

The experimental data supporting the findings of this research will be available upon reasonable request.
